# A Cross-Sectional Study of Compositional and Functional Profiles of Gut Microbiota in Sardinian Centenarians

**DOI:** 10.1128/mSystems.00325-19

**Published:** 2019-07-09

**Authors:** Lu Wu, Tiansheng Zeng, Angelo Zinellu, Salvatore Rubino, David J. Kelvin, Ciriaco Carru

**Affiliations:** a Division of Immunology, International Institute of Infection and Immunity, Shantou University Medical College, Shantou, Guangdong, China; b Department of Biomedical Sciences, University of Sassari, Sassari, Italy; c Department of Microbiology and Immunology, Dalhousie University, Halifax, Canada; University of Pennsylvania

**Keywords:** centenarian, gut microbiota, longevity, metagenomic sequencing

## Abstract

Sardinia, Italy, has a high prevalence of residents who live more than 100 years. The reasons for longevity in this isolated region are currently unknown. Gut microbiota may hold a clue. To explore the role gut microbiota may play in healthy aging and longevity, we used metagenomic sequencing to determine the compositional and functional differences in gut microbiota associated with populations of different ages in Sardinia. Our data revealed that the gut microbiota of both young and elderly Sardinians shared similar taxonomic and functional profiles. A different pattern was found in centenarians. Within the centenarian group, the gut microbiota was correlated with the functional independence measurement of the host. Centenarians had a higher diversity of core microbiota species and microbial genes than those in the young and elderly. We found that the gut microbiota in Sardinian centenarians displayed a rearranged taxonomic pattern compared with those of the young and elderly, featured by depletion of Faecalibacterium prausnitzii and Eubacterium rectale and enriched for Methanobrevibacter smithii and Bifidobacterium adolescentis. Moreover, functional analysis revealed that the microbiota in centenarians had high capacity for central metabolism, especially glycolysis and fermentation to short-chain fatty acids (SCFAs), although the gut microbiota in centenarians was low in genes encoding enzymes involved in degradation of carbohydrates, including fibers and galactose.

**IMPORTANCE** The gut microbiota has been proposed as a promising determinant for human health. Centenarians as a model for extreme aging may help us understand the correlation of gut microbiota with healthy aging and longevity. Here we confirmed that centenarians had microbiota elements usually associated with benefits to health. Our finding of a high capacity of glycolysis and related SCFA production represented a healthy microbiome and environment that is regarded as beneficial for host gut epithelium. The low abundance of genes encoding components of pathways involved in carbohydrate degradation was also found in the gut microbiota of Sardinian centenarians and is often associated with poor gut health. Overall, our study here represents an expansion of previous research investigating the age-related changes in gut microbiota. Furthermore, our study provides a new prospective for potential targets for gut microbiota intervention directed at limiting gut inflammation and pathology and enhancing a healthy gut barrier.

## Abstract (precis)

The gut microbiota has been proposed as a promising determinant for human health. Centenarians as a model for extreme aging may help us understand the correlation of gut microbiota with healthy aging and longevity. Here we confirmed that centenarians had microbiota elements usually associated with benefits to health. Our finding of a high capacity of glycolysis and related SCFA production represented a healthy microbiome and environment that is regarded as beneficial for host gut epithelium. The low abundance of genes encoding components of pathways involved in carbohydrate degradation was also found in the gut microbiota of Sardinian centenarians and is often associated with poor gut health. Overall, our study here represents an expansion of previous research investigating the age-related changes in gut microbiota. Furthermore, our study provides a new prospective for potential targets for gut microbiota intervention directed at limiting gut inflammation and pathology and enhancing a healthy gut barrier.

## INTRODUCTION

Longevity is a complex biological phenotype determined by genetic, epigenetic, and environmental factors such as diet, lifestyle, and even geographic location (1–4). These factors have also been shown to affect the gut microbiota in humans (5–8). It has been demonstrated that gut microbiota is tightly linked to human health and disease (9). There is evidence showing that the gut microbiome contributes to the regulation of host life span in animal models, such as Caenorhabditis elegans, Nothobranchius furzeri (turquoise killifish), Heterocephalus glaber (naked mole-rat) and *Drosophila* (10–13). Moreover, a longitudinal study has also found association between the distinct metabolomic signatures and longevity of humans (14). Thus, gut microbiota may also modulate human longevity by affecting the host metabolism. Manipulating the gut microbiota with diet intervention and calorie restriction (CR) has potential therapeutic applications for pro-healthy aging intervention (4, 15).

Several groups have used centenarians as a model to study aging and gut microbiota (16–22), with most studies focusing on the compositional features of gut microbiota. Even though a few studies have examined the metabolic function of gut microbiota in centenarians (18, 22, 23), metagenomic analysis of metabolic functions has yet to be fully explored in diverse populations from various geographic regions. The Mediterranean island of Sardinia is well-known for the unique isolated genomic background and the high prevalence of centenarians (2, 24, 25). The high prevalence of centenarians, consistent lifestyle, and low immigration rates make Sardinia an ideal geographic area for the study of longevity. However, little is known about how the Sardinian environment and genetic factors influence the gut microbiota in Sardinian centenarians (26–29). Surveying the gut microbiota in Sardinian centenarians may also expand our understanding of longevity across global populations.

Here we performed a cross-sectional survey of the gut microbiota in the longevity-prone population in Sardinia by metagenomic sequencing. In our study, we recruited 65 subjects, divided into three age groups: the young, elderly, and centenarians. We obtained the taxonomic composition and functional annotation of the gut microbiota in the different age groups. We also correlated health status with gut microbiota in centenarians.

## RESULTS

### Characterization of gut microbiota compositional profiles in the three age groups.

To obtain the taxonomic compositional and functional profiles of gut microbiota in the Sardinian population, we recruited a cohort of three age groups: healthy young (*n* = 19), healthy elderly (*n* = 25), and centenarians (*n* = 21). The clinical characteristics are shown in Table 1 and Table S1 in the supplemental material. A total of 59 qualified stool samples were used to extract microbial DNA for DNA library construction and shotgun metagenomic sequencing. On average, 5.8 Gb data (approximately 41.3 million high-quality clean reads) were generated per sample. Human contamination was removed (on average, up to 14% of the total reads) before further processing. The taxonomic compositional profile was generated using MetaPhlAn2 (30). We verified our results using the IGC database (31). The workflow is shown in Fig. S1 in the supplemental material.

**TABLE 1:** Demographical and clinical characteristics in the three age groups^*a*^

Parameter	Value for parameter, mean ± SD (range), for the following age group:		
	Centenarians (*n* = 19)	Elderly (*n* = 23)	Young (*n* = 17)
Age (yr)	101.8 ± 1.4 (99–107)	76.7 ± 5.9 (68–88)	25.5 ± 3.5 (21–33)
Male (%)	23.50	43.40	58.8
Weight (kg)	57.1 ± 5.7 (43–73)	68.7 ± 14.3 (42–103)	63.2 ± 3.5 (44–95)
BMI (kg/m^2^)	23.5 ± 2.1 (17.9–28.1)	25.9 ± 4.1 (19.5–36.9)	22.8 ± 3.7 (16.1–40.1)
MMSE (0,30)	15.8 ± 6.7 (5–26)	26.6 ± 3.0 (22–30)	NA
MNA (0,30)	18.9 ± 3.7 (8–26)	24.1 ± 2.0 (18–28)	24.6 ± 2.1 (20.5–28)
FIM (0,126)	77.5 ± 21.1 (31–123)	123.7 ± 1.9 (119–126)	NA

a The total number of subjects is 59 excluding individuals with unqualified stool samples (*n* = 6). BMI, body mass index; MMSE, mini-mental state examination; MNA, mini-nutritional assessment; FIM, functional independence measure; NA, not available.

10.1128/mSystems.00325-19.1 TABLE S1 Full demographic and clinical information for each subject. Download Table S1, DOCX file, 0.04 MB. Copyright © 2019 Wu et al. 2019 Wu et al. This content is distributed under the terms of the Creative Commons Attribution 4.0 International license.

10.1128/mSystems.00325-19.4 FIG S1 Workflow to analyze gut microbiota in the three age groups. Download FIG S1, EPS file, 2.1 MB. Copyright © 2019 Wu et al. 2019 Wu et al. This content is distributed under the terms of the Creative Commons Attribution 4.0 International license.

The gut microbiota compositional profiles for the three age groups are shown in Data Set S1 in the supplemental material. At the phylum level, the gut microbiota is dominated by *Firmicutes*, *Bacteroidetes*, *Actinobacteria*, and *Proteobacteria*, which is in agreement with a previous study using a different cohort (Fig. S2A and B) (32). A lower abundance of *Firmicutes* was found in the centenarian cohort compared with that in the elderly cohort (Kruskal-Wallis followed by Dunn’s post-hoc multiple-comparison test, χ^2^ = 12.893, df = 2, *P* value = 0.0016; Fig. S2C). *Proteobacteria* was enriched in centenarians compared to that in the young and elderly (Kruskal-Wallis followed by Dunn’s post-hoc multiple-comparison test, χ^2^ = 9.0686, df = 2, *P* value = 0.0107; Fig. S2C). As the most abundant two phyla in the gut, the *Firmicutes/Bacteroidetes* ratio (F/B ratio) is often used as an index for the structure of gut microbiota; we found that the F/B ratio was significantly lower in the centenarians than in the elderly (Kruskal-Wallis followed by Dunn’s post-hoc multiple-comparison test; Fig. S2D).

10.1128/mSystems.00325-19.3 DATA SET S1 Taxonomic composition and functional profiles of the gut microbiota for each subject. Sheet 1, phylum level taxonomic composition profiles; sheet 2, genus level taxonomic composition profiles; sheet 3, species level taxonomic composition profiles; sheet 4, gene pathway functional profiles. Download Data Set S1, XLSX file, 0.4 MB. Copyright © 2019 Wu et al. 2019 Wu et al. This content is distributed under the terms of the Creative Commons Attribution 4.0 International license.

10.1128/mSystems.00325-19.5 FIG S2 Variations in the compositional structures of gut microbiota among the three age groups at the phylum level. The dominant phyla were compared by two different analysis methods for metagenomic sequencing. (A) Mean relative abundance for each phylum in three age groups surveyed by the HUMANN2 method; (B) mean relative abundance for each phylum in three age groups surveyed by IGC method; (C) boxplot of the relative abundance of the dominant phyla in three age groups (HUMANN2 method); (D) boxplot of the of F/B ratio for each individual in the three age groups. Variation was tested by Kruskal-Wallis followed by Dunn’s post-hoc multiple-comparison test. Symbols: *, *P* < 0.05; **, *P* < 0.01. Download FIG S2, EPS file, 1.3 MB. Copyright © 2019 Wu et al. 2019 Wu et al. This content is distributed under the terms of the Creative Commons Attribution 4.0 International license.

**FIG 1:**
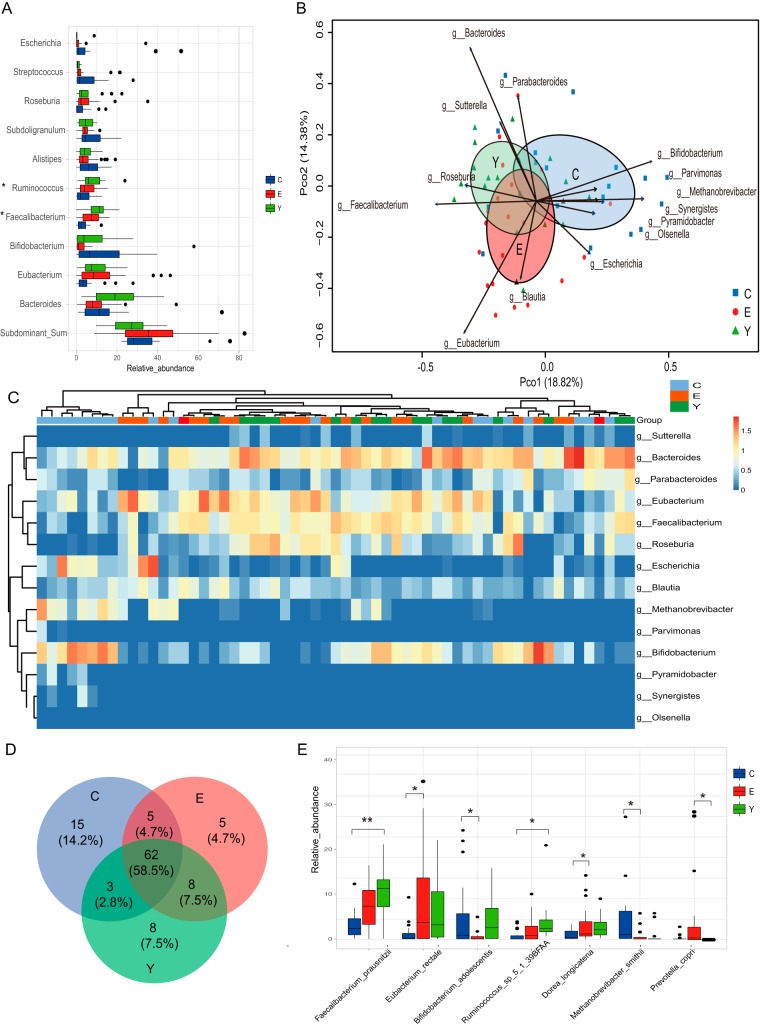
Dynamic signature of gut microbiota components in the three age groups. (A) Relative abundance of the top 10 genera in the three age groups. Other low-abundant genera are summed into one group labeled “Subdominant_Sum.” The genera with significantly different distributions among the three age groups detected by ANOVA test (*P* < 0.05) are marked with an asterisk. (B) PCoA based on the Bray-Curtis distance derived from the relative abundance of the genus was plotted for the gut microbiota composition at the genus level. Ellipses with 95% confidence around the centroid of each age group are plotted in PCoA; the age groups are labeled C for the centenarian group, E for the elderly group, and Y for the young group. The genera that significantly correlated with the ordination in PCoA are shown as arrows (permutation test, *P* < 0.01), with the length of the arrow indicating the goodness of fit statistic, squared correlation coefficient. (C) Heatmap of the relative abundance of genera that are significantly correlated with the separation of the samples in PCoA in the three age groups. The base 10 logarithm of relative abundance was used as input, and complete linkage clustering was used. The distance matrix was created by the “Pearson” method. (D) Core microbiota species distribution in the three age groups. The core microbiota is defined as the species shared by more than 50% of the individuals in each age group. (E) Relative abundance of the species that showed significantly different distributions in the three age groups (Kruskal-Wallis followed by Dunn’s post-hoc multiple-comparison test, *P* < 0.05). Values that are significantly different are indicated by a bar and asterisks as follows: *, *P* < 0.05; **, *P* < 0.01.

To explore the gut microbiota composition in detail, we determined the relative abundance of the dominant genera in the gut microbiota for the three age groups (Fig. 1A). We observed that the relative abundance of the dominant genus showed considerable diversity among the three age groups. A lower relative abundance for *Faecalibacterium*, *Ruminococcus*, *Corprococcus*, and *Dorea* was observed in the centenarians compared with the abundances found in the young and elderly (analysis of variance [ANOVA] test, *P* value < 0.05; Fig. S3A) *Methanobrevibacter*, a dominant *Archaea* in the human gut ecosystem, as well as the subdominant genera *Pyramidobacter* and *Desulfovibrio*, were enriched in the centenarians (ANOVA test, *P* value < 0.05).

10.1128/mSystems.00325-19.6 FIG S3 Variations in the compositional structures of gut microbiota among the three age groups at the genus level. (A) Boxplot of the relative abundance of the dominant genera that are different in the three age groups. Only the dominant genera with significant difference (*P* value < 0.05) between age groups detected by ANOVA followed by Tukey-Kramer multiple-comparison test are shown. Significance symbols: *, *P* < 0.05; **, *P* < 0.01; ***, *P* < 0.001. Relative abundance of the dominant genera show that the similarity of the individuals is driven by the relative abundance of the dominant genera. PCoA based on the Bray-Curtis distance derived from the relative abundance of the genus was plotted for the gut microbiota composition. The size of the circle presents the abundance of the genus in each individual. (B) *Bacteroides*; (C) *Faecalibacterium*; (D) *Bifidobacterium*; (E) *Eubacterium*. (F) The boxplots show the relative abundance of *Bifidobacterium* and *Methanobrevibacter* in the Sardinia and Emilia Romagna cohorts (the Emilia Romagna cohort was studied by 16S rRNA sequencing). S_C/E/Y denotes the Sardinian centenarians/elderly/young, R_SC/C/E/Y denotes the Emilia Romagna semisupercentenarians/centenarians/elderly/young. Download FIG S3, EPS file, 1.7 MB. Copyright © 2019 Wu et al. 2019 Wu et al. This content is distributed under the terms of the Creative Commons Attribution 4.0 International license.

To further investigate the similarity of the community structure (at the genus level) of the gut microbiota for each individual among the three age groups, we used principal-coordinate analysis (PCoA) based on the Bray-Curtis distance of the microbial community at the genus level to visualize the distribution and clustering of the subjects. We found that the three age groups clustered separately (Fig. 1B). The elderly group cluster overlapped with the young group but showed a slight shift, while the centenarian group had some centenarians with profiles similar to those of the young and elderly, but the cluster shifted in a different direction from that of the elderly. Analysis of similarities (ANOSIM) test using Bray-Curtis distance revealed that no significant difference in the composition of gut microbiota at the genus level was evident between the young and elderly (R value = −4.602e−05, *P* value = 0.464). However, significant differences between centenarians and the young were observed (R value = 0.1792, *P* value = 0.001); significant differences between the centenarians and the elderly (R value = 0.1707, *P* value = 0.001) were also observed. Multiple response permutation procedure (MRPP) analysis revealed that the delta of the young was 0.65, while that of the elderly was 0.74 and that of the centenarians was 0.76, showing that within-group distance is larger in the elderly and centenarian groups, consistent with the size of the ellipses in the PCoA. MRPP also revealed significant differences in the gut microbiota composition at the genus level among the three age groups (*P* = 0.001, A = 0.03). We observed that the distribution of the individuals in the PCoA was driven by dominant genera (see Fig. S3B to E in the supplemental material). The genera significantly contributing to the ordination of the samples are shown in Fig. 1B (EnvFit analysis by permutation test, *P* value < 0.01). *Faecalibacterium*, *Bacteroides*, *Roseburia*, *Sutterella*, and *Parabacteroides* are positively correlated and significantly contribute to the cluster of the young group, while *Eubacterium* and *Blautia* are positively correlated and significantly contribute to the cluster of the elderly. The enrichment of *Bifidobacterium*, *Methanobrevibacter*, *Pyramidobacter*, *Synergistes*, and *Escherichia* were detected and positively correlated with the cluster of centenarians. Interestingly, the enrichment of *Bifidobacterium* and *Methanobrevibacter* observed in Sardinian centenarians was also found in centenarians from Emilia Romagna, Italy (16) (Fig. S3F). The heatmap of the relative abundance of the genera that significantly correlate with the cluster of the age groups is displayed in Fig. 1C. Noticeably, eight centenarians form a cluster in the hierarchical cluster with a high abundance of *Bifidobacterium*, *Methanobrevibacter*, and *Escherichia* (Fig. 1C).

We next investigated the taxonomic composition at the species level and found the α diversity of gut microbiota (evaluated by Shannon diversity index and species richness) was not significantly different among age groups (see Fig. S4A and B in the supplemental material, Kruskal-Wallis test, *P* > 0.05). “Core microbiota” is used to identify and describe the key microorganisms that are considered to be stable and permanent within a community (33). Here the core microbiota is defined as microbes that are present in at least 50% of the samples at the species level. In our data set, we found that core microbiota in centenarians showed a higher species richness compared with the young and elderly (Fig. 1D). The relative abundance of the centenarian-specific core microbe distribution in all three age groups is shown in Fig. S4C in the supplemental material. We observed the enrichment of several species belonging to *Bacteroides*, *Bifidobacterium*, *Clostridium*, *Erysipelotrichaceae*, and *Lactobacillus* in centenarians. The relative abundance of species with significant differences between the three different age groups is shown in Fig. 1E (Kruskal-Wallis followed by Dunn’s post-hoc multiple-comparison test, *P* < 0.05). We observed the low abundance of Faecalibacterium prausnitzii, Eubacterium rectale, and *Ruminococcus sp_5_1_39BFAA* but a high abundance of Bifidobacterium adolescentis and Methanobrevibacter smithii in the centenarian gut microbiota (Fig. 1E).

10.1128/mSystems.00325-19.7 FIG S4 Variations of the compositional structures of gut microbiota among the three age groups at the species level. (A) Boxplot of Shannon diversity index in the three age groups; (B) boxplot of species richness in the three age groups; (C) boxplot of the relative abundance of centenarian unique core microbiota in the three age groups. Variations are detected by the Kruskal-Wallis test. Download FIG S4, EPS file, 1.2 MB. Copyright © 2019 Wu et al. 2019 Wu et al. This content is distributed under the terms of the Creative Commons Attribution 4.0 International license.

### Potential functional annotations of gut microbiota in the three age groups.

**FIG 2:**
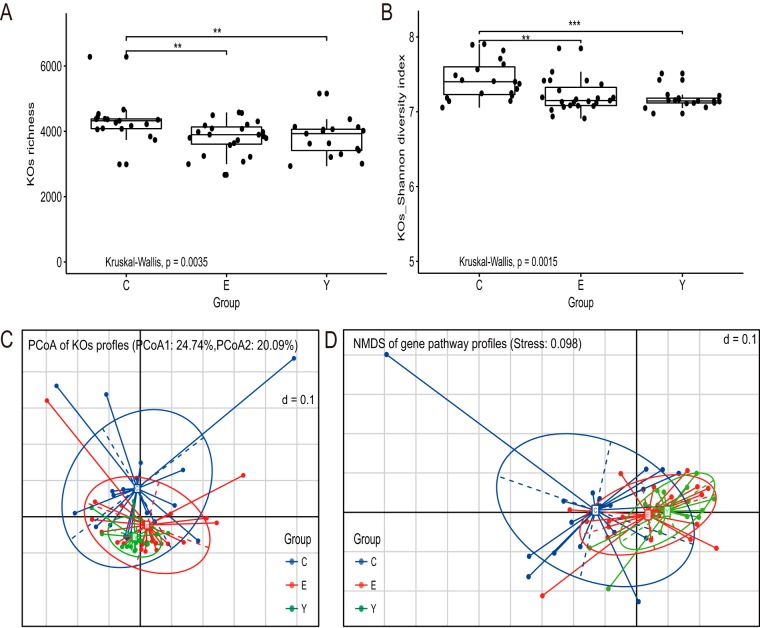
Diversity of potential functional profiles of gut microbiota in the three age groups. (A and B) Boxplot of the distribution of KO richness (A) and of the distribution of KO Shannon diversity index (B). Variation between different age groups was detected by Kruskal-Wallis followed by Dunn’s post-hoc multiple-comparison test. Statistical significance symbols: *, *P* < 0.05; **, *P* < 0.01; ***, *P* < 0.001. (C and D) Dissimilarities of the functional profile of gut microbiota among the three age groups based on the Bray-Curtis distance derived from the relative abundance of KOs using PCoA (C) or derived from the relative abundance of gene pathways using NMDS (D).

To determine whether the metabolic potential of gut microbiota shows variation within different age groups, metagenomic sequencing data were processed by the Humann2 pipeline. The relative abundance of gene families and gene pathways was obtained. We detected 384,425 gene families assigned to 1,924 species. A total of 463 gene pathways were rebuilt. We compared the mean relative abundance for each of the KEGG orthology (KO) between different age groups (Fig. S5). We observed that the majority of KOs present in gut microbiota were in low proportions (<0.02%) (Fig. S5). Furthermore, the young and elderly shared similar abundance patterns (Fig. S5B). Compared with elderly and young, the centenarians have a lower abundance of most of the dominant KOs (Fig. S5A and C). The Shannon diversity of KOs and richness of KOs (defined as the number of KOs detected within each subject) was significantly higher in the centenarian group than in the young and elderly groups, while in the young and elderly groups, they were not significantly different (Fig. 2A and B, Kruskal-Wallis followed by Dunn’s post-hoc multiple-comparison test).

10.1128/mSystems.00325-19.8 FIG S5 Relative proportion of all KEGG orthologs detected within gut microbiota in the three age groups. (A) Centenarians compared with elderly; (B) elderly compared with young; (C) centenarians compared with young. Points on either side of the gray dashed *y* = *x* line are enriched in one of the two groups. Welch’s two-sided *t* test was used to determine whether the observed difference was significant. Confidence intervals for each KO are displayed and are calculated using the Wilson score method. Download FIG S5, EPS file, 2.3 MB. Copyright © 2019 Wu et al. 2019 Wu et al. This content is distributed under the terms of the Creative Commons Attribution 4.0 International license.

Gut microbiota functional similarities were assessed among individuals by PCoA based on the Bray-Curtis distance derived from the relative abundance of KOs, as shown in Fig. 2C and by nonmetric multidimensional scaling (NMDS) based on the Bray-Curtis distance derived from the relative abundance of gene pathways, as shown in Fig. 2D. These analyses demonstrated that, consistent with the taxonomic profiles, the interindividual differences within each age group are larger in the elderly and centenarian groups (Fig. 1B). The elderly group shared similar functional profiles with the young group but differed strikingly from the centenarian group (Fig. 2C and D). The ANOSIM test, using Bray-Curtis distance on the relative abundance of KOs, also revealed that no significant difference in the KO profiles between the young and elderly were observed (R value = −0.001716, *P* value = 0.465). However, a significant difference between the centenarians and the young (R value = 0.1406, *P* value = 0.003), and between the centenarians and the elderly, was observed (R value = 0.1247, *P* value = 0.004).

**FIG 3:**
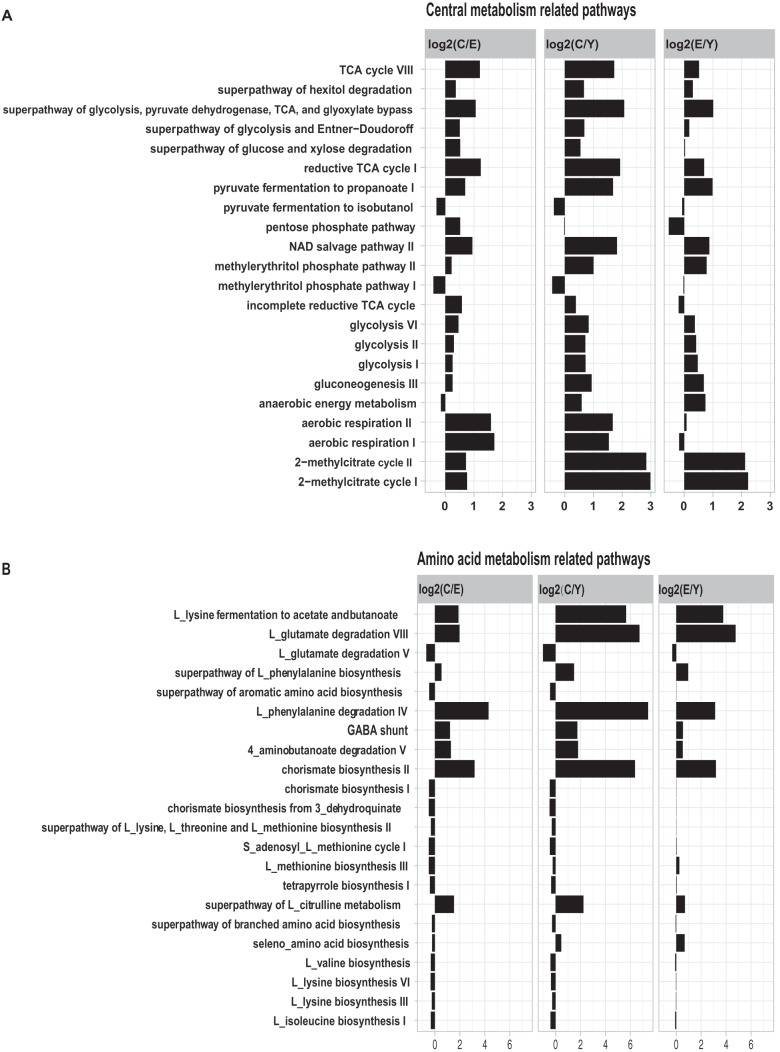

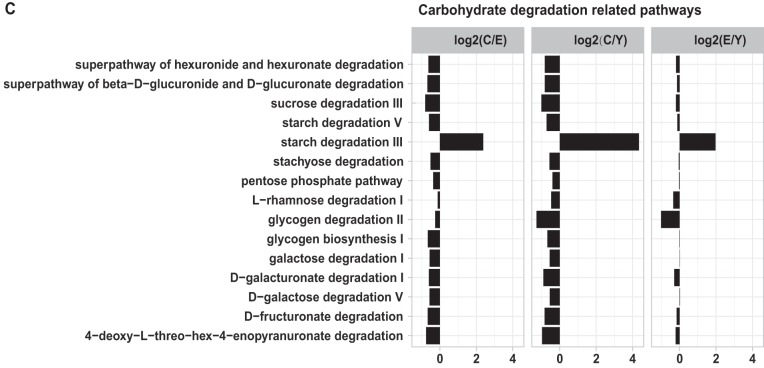
Functional signatures of gut microbiota in the three age groups. Relative abundance of the gene pathways that are significantly different in the three age groups (ANOVA, *P* < 0.05). The centenarian group compared with the elderly group (C versus E [C/E]), the centenarian group compared with the young group (C versus Y [C/Y]), and the elderly group compared with the young group (E versus Y [E/Y]) are shown in each panel. The length of the bar indicates the base 2 logarithm value of the relative abundance ratio for each age group; 0 represents equal abundance in the two groups. Gene pathways are grouped in related pathways: central metabolism-related pathways (A), amino acid metabolism-related pathways (B), and carbohydrate degradation-related pathways (C).

Although nearly all the gene pathways we detected were shared by all the age groups, the dominant pathways were conserved in all individuals (see Data Set S1 in the supplemental material). For example, the gene pathways for nucleotide biosynthesis and cell wall biosynthesis were highly abundant in all age groups. However, 115 pathways of the 463 pathways had significant variation among the three age groups (ANOVA test, *P* < 0.05). Regrouped into four main metabolic functional classes, the relative abundance of the gene pathways in the three age groups is shown in Fig. 3 and Fig. S6 in the supplemental material.

10.1128/mSystems.00325-19.9 FIG S6 Relative abundance of the KOs and gene pathways within the gut microbiota in the three age groups. (A) PTS system transporters; (B) F420; (C) coenzyme M. Variations are detected by Kruskal-Wallis test. Download FIG S6, EPS file, 1.8 MB. Copyright © 2019 Wu et al. 2019 Wu et al. This content is distributed under the terms of the Creative Commons Attribution 4.0 International license.

Gene pathways involved in central metabolism, including glycolysis, pentose phosphate pathways, and the tricarboxylic acid (TCA) cycle, as well as anaerobic respiration, had a higher abundance in the elderly group compared with that of the young group, and the abundance was even greater in the centenarian group than in the elderly group (Fig. 3A, ANOVA test, *P* < 0.05). Additionally, in the gut microbiota in centenarians, we detected a high abundance of KOs for the phosphotransferase system (PTS) and the major facilitator superfamily (MFS) system transporters, which can facilitate the transfer of carbohydrates into the cytoplasm of bacteria (see Fig. S6A in the supplemental material). Noticeably, several pathways that were related to the metabolism of SCFAs were enriched in the centenarian group, for example, the pathways involved in pyruvate fermentation to propionate I and 2−methylcitrate cycle I and II (propionate degradation) (Fig. 3A). The anaerobic energy metabolism pathway which is involved in fermentation to SCFAs (propanoate and acetate) was also higher in the centenarians compared to that in the young (Fig. 3A). The abundance of aerobic respiration pathways was similar for the elderly and young groups but remarkably higher in the centenarians (Fig. 3A).

Protein and amino acid metabolism-related pathways are shown in Fig. 3B (ANOVA test, *P* < 0.05). As expected, gut microbes in the centenarians but not the healthy elderly exhibited a lower abundance in most of the amino acid biosynthesis pathways compared with that in the young group. For instance, l-lysine-, l-isoleucine-, and l-methionine-related pathways are lower in centenarians. Additionally, certain pathways related to the aromatic compounds are enriched in the elderly compared with those in the young, yet enriched to a greater extent in the centenarians. These pathways include the l-phenylalanine metabolism-related pathways and the chorismate biosynthesis II pathway. Interestingly, we found that SCFA production via fermentation of amino acids such as l-lysine fermentation to acetate and butanoate and l-glutamate degradation VIII (to propanoate), as well as GABA shunt pathway and 4-aminobutanoate degradation V pathway (to butyrate), were also dramatically enriched in the centenarian group (Fig. 3B).

Our results also revealed that the relative abundance of the pathways related to carbohydrate degradation was similar for the elderly and young groups but significantly lower in the centenarians, with the exception of the starch degradation III pathway which is utilized by *Archaea* (Fig. 3C). Interestingly, the galactose degradation-related pathways were also remarkably low in the centenarians (Fig. 3C).

Gene pathways related to vitamin metabolism are shown in Fig. S7 in the supplemental material (ANOVA test, *P* < 0.05). We found the centenarian group displayed a significant enrichment of menaquinone (vitamin K2) gene pathways compared with the elderly group in gut microbiota. We further noticed that the menaquinone-related pathway abundance also showed enrichment in the elderly group compared with that in the young group. Moreover, the riboflavin (vitamin B_2_) synthesis pathway was also highly enriched in the centenarians. The gene families specific for *Archaea*, such as coenzyme M and F420, were detected as remarkably enriched in the centenarian group as well (see Fig. S6B and C in the supplemental material). The gene pathway abundance of thiamine synthesis (vitamin B_1_) was lower in the elderly and centenarian groups.

10.1128/mSystems.00325-19.10 FIG S7 Functional signatures of gut microbiota in the three age groups. Relative abundance of the vitamin metabolism-related gene pathways that are significantly different in the three age groups (ANOVA test, *P* value < 0.05). The centenarian group compared with the elderly group (C versus E), and the centenarian group compared with the young group (C versus Y), and the elderly group compared with the young group (E versus Y) are shown in each panel. The length of the bar indicates the base 2 logarithm value of the relative abundance ratio between each age group. Download FIG S7, EPS file, 0.7 MB. Copyright © 2019 Wu et al. 2019 Wu et al. This content is distributed under the terms of the Creative Commons Attribution 4.0 International license.

### Correlation between gut microbiota and health status.

**FIG 4:**
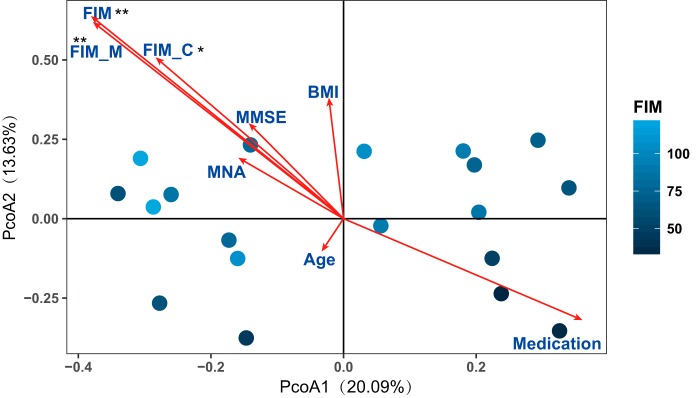
Gut microbiota in centenarians correlates with clinical parameters. PCoA based on the Bray-Curtis distance derived from the relative abundance of species was plotted for the bacterial microbiota composition of centenarians at the species level. Each circle represents the value for an individual centenarian in PCoA, with the FIM index for the centenarian indicated by the color. Clinical parameters, including FIM (cognitive FIM [FIM_C] and motor FIM [FIM_M]), MMSE, MNA, BMI, age, and medication were used as factors to show correlation with the ordination configuration by EnvFit analysis. “Medication” indicates the number of drug types taken daily. The length of lines indicates the goodness of fit statistic, the squared correlation coefficient. The permutation test was used to test the significance of the fitness; the number of permutation was 999. Significance symbol: *, *P* < 0.05; **, *P* < 0.01.

The demographic and clinical values for 59 subjects within the three age groups are shown in Table 1. On average, the entenarians in our cohort scored poorly for diverse health parameters, including mini-mental state examination (MMSE), mini nutritional assessment (MNA), and functional independence measure (FIM), compared with the elderly, whose scores were similar to those of the young (see Table S2 in the supplemental material). Health parameters may act as covariates and associate with the host gut microbiota composition. To explore the significance of health covariates, EnvFit analysis was used to determine the correlation of the health parameters with gut microbiota in centenarians (Fig. 4, permutation test, *P* value < 0.05; ordination was performed using PCoA based on Bray-Curtis distance of relative abundance of species). We found that FIM covariates were significantly associated with the species level of bacterial community profiles in centenarians. The length of the FIM arrow indicated that the FIM score might explain the greatest amount of variance between individuals in the PCoA. This further suggested that centenarians with similar FIM scores tend to have similar gut microbiota composition. We also observed that age was not significantly associated with FIM, which further suggested that health in centenarians was not related to chronological age. The MNA was positively related to FIM, emphasizing the potential importance of diet for maintaining healthy aging.

10.1128/mSystems.00325-19.2 TABLE S2 Statistical variation between the demographic and clinical information in different age groups. Download Table S2, DOCX file, 0.03 MB. Copyright © 2019 Wu et al. 2019 Wu et al. This content is distributed under the terms of the Creative Commons Attribution 4.0 International license.

## DISCUSSION

The gut microbiota has been proposed as an important determinant of human health (9, 34). Modulation of the gut microbiota is a rapidly emerging field of study and holds promise for impacting longevity and healthy aging (35). Here we utilized metagenomic sequencing to address the compositional and functional features of the gut microbiota in centenarians and the young and elderly in Sardinia, Italy. The island of Sardinia is regarded as an ideal geographic location to study longevity due to its isolated nature, a high incidence of centenarians, a relatively homogeneous population, lifestyle, and the Mediterranean diet (2, 24, 25, 27). Our study here sought to identify the gut microbiota community structures at the species level. Since taxonomic composition alone does not necessarily provide a complete understanding of community function for the gut microbiota, we conducted functional analysis of the gut microbiota to explore the potential metabolic role of gut microbiota in centenarians. Our results and analysis led us to several main findings and conclusions. We found that the gut microbiota in Sardinian centenarians displayed a rearranged taxonomic pattern compared with the gut microbiota of the young and elderly, featured by depletion of F. prausnitzi and E. rectale, and enriched for M. smithii and B. adolescentis. Moreover, we found that the gut microbiota in Sardinian centenarians have a high capacity for central metabolism, especially glycolysis and short-chain fatty acid production, although we found a poor capacity for degradation of carbohydrates. Even though we found evidence of genes encoding components of several pathways that may provide health benefits, there are indications that other microbiota features and function in centenarians may contribute to inflammation and gut barrier problems.

The compositional features of the gut microbiota for centenarians have been described previously using quantitative PCR (qPCR), microarray, and 16S rRNA sequencing as well as metagenomic sequencing (16, 19–23, 36). We have extended these earlier studies by identifying the taxonomic composition at the species level and functional composition at the pathway level using metagenomic sequencing and analysis. We found that the taxonomic composition in gut microbiota in the young and elderly is not statistically different. These findings are consistent with those of a study that indicates the gut microbiota of healthy aged Chinese are similar to those of the healthy young (37). Furthermore, we found that the gut microbiota composition in centenarians is statistically different from the gut microbiota in the young and elderly in Sardinia.

A distinctive gut microbiota structure in centenarians has also been demonstrated in previous studies from Guangxi, China; Emilia Romagna, Italy; and Manipur, India (16, 20, 22, 36). Among the observed gut microbiota features in centenarians found in these studies and in our study, some features were noted to be unique to defined populations from specific geographic locations; for example, the enrichment of *Methanobrevibacter* and *Bifidobacterium* in centenarians detected in our cohort was also found in the Emilia Romagna, Italy cohort (16). However, the enrichment of *Methanobrevibacter* and *Bifidobacterium* was not found in the Sichuan and Guangxi cohorts in China or in a national Japanese cohort and a Manipur, Indian cohort (19–22). Although *Akkermansia* was enriched in the centenarians from Emilia Romagna, Italy, and Manipur, India (16, 22), it was found in low abundance in centenarians in our cohort and in the Guangxi, China cohort (20). Overall, the previous studies on gut microbiota in centenarians reveal that the gut microbiota in long-living peoples have diverse features, which may be a consequence of various adaptations of the gut microbiota to aging in different geographical locations where different populations are under the influence of diverse genetic, dietary, physiological, and environmental conditions. It must also be pointed out that different methodologies for recruitment of subjects, collection and processing the samples, and analysis of data may contribute to differences observed between different centenarian populations. Nevertheless, we also observed several features that appear to be more universal, such as the low abundance of *Faecalibacterium* in centenarians (16, 20–22, 36), as well as the enrichment of *Methanobrevibacter* and *Desulfovibrio*, which both belong to electron acceptor species. *Methanobrevibacter* and *Desulfovibrio* were found enriched in our and other centenarian cohorts and enriched in the long-living naked mole-rat animal model (12, 16, 21, 22). Furthermore, Escherichia coli, which was reported to be enriched in centenarians from Emilia Romagna, Italy; Guangxi, China; and Japan, was also enriched in our cohort (20, 21, 36). The shared microbiota features and the impact on centenarian heath are not clear at this time.

Beyond taxonomic composition, our metagenomic data provided the opportunity to functionally annotate the gut microbiota. We examined the age-related metabolic functional variations in the gut microbiota from the young, elderly, and centenarians and found that the gut microbiota is functionally similar between the young and elderly but is different for centenarians. Biodiversity of the gut microbiota community is frequently used to indicate the function and stability of the gut ecosystem (38, 39). The significantly higher α diversity of KOs in centenarians compared with the microbiota from the young and elderly indicates functional diversity and plasticity in the centenarian gut microbiota. Our study not only highlighted the specific metabolic patterns regarding carbohydrate metabolism and central metabolism of gut microbiota in centenarians but also revealed the specific distribution of several metabolic pathways that have a critical role in host health and aging.

In Sardinian centenarians, we found an enrichment of genes encoding components of glycolysis-related pathways in the gut microbiota. The high abundance of sugar membrane transporters in the centenarian gut microbiota indicates the potential for increased microbe uptake of simple sugars in the gut lumen, providing the initial substrate for glycolysis. Previous studies have indicated that *Lactobacillus* and *Escherichia* have high glycolysis capability (40), and in our study, the enrichment of gene pathways related to glycolysis correlated with the enrichment of *Lactobacillus* and *Escherichia* in centenarians. Previous studies have shown that *M. smithii* can cooperate with the *Bacteroides* to enhance fermentation (41). The electron acceptors *M. smithii* and *Desulfovibrio* were found to be abundant in centenarians by us as well as others (16, 21, 22). The presence of these bacteria may aid in the elimination of fermentation products that limit glycolysis. These previous studies and our findings sugges thatt glycolysis may be enhanced in centenarian populations.

Glycolysis-derived pyruvate is a key metabolite for biosynthesis of SCFAs from carbohydrate fermentation and bacterial cross-feeding (42). Pyruvate is the major precursor of fermentation products for the synthesis of the three major SCFAs, acetate, propionate, and butyrate (42). SCFA formation can also take place from organic acid and amino acid metabolism (43). SCFAs have been shown to have important functions in the human host; for example, they serve as energy substrates for colonocytes, and the oxidation of SCFAs by colonocytes plays a critical role in maintaining luminal oxygen levels (42, 44, 45). SCFAs also can protect the mucous layer and enhance the secretion of mucus (46). SCFAs also can act as ligands for G-protein-coupled-receptors (GPCRs), directly activating GPR43 and GPR41 to release peptide YY (PYY) and glucagon-like peptide 1 (GLP-1), which in turn play important roles in the regulation of food intake and insulin secretion (47). SCFAs also act as signaling molecules by inhibiting histone deacetylases, which are related to anti-tumor and anti-inflammation functions by regulating macrophages, dendritic cells, regulatory T cells (Tregs), and B-cell IgA production as well as cytokine expression in T cells (47–52). Thus, SCFAs are important for the maintenance of gut health and homeostasis. Enhanced glycolysis may lead to increased production of SCFAs. For example, genes encoding components of the pyruvate fermentation to propionate pathway are enriched in our centenarian cohort. Moreover, we found the enrichment of pathways that produce SCFAs by protein and amino acid fermentation in the gut microbiota of centenarians. Specifically, we found significant enrichment of the 2-methylcitrate cycle pathway, the l-lysine fermentation to acetate and butanoate pathway, and the l-glutamate degradation VIII pathway in the gut microbiota in centenarians. In addition, pathways involved in butyrate production, such as GABA shunt and 4-aminobutanoate degradation V were also enriched in the gut microbiota in centenarians. Overall, the gut microbiota in centenarians appear to have the potential of enhancing SCFA production. It is noteworthy that previous studies have indicated that the amount and relative abundance of SCFA may be considered biomarkers of a healthy status (53, 54). Interestingly, previous studies also have demonstrated higher levels of SCFAs in stool samples from centenarians than in stool samples from the elderly (4, 22). Thus, the enhanced capability of SCFA production in centenarians suggests that the SCFAs may enhance the gut barrier function and reduce inflammation with aging. However, the hypothesis of enhanced SCFA production in Sardinian centenarians will be explored in the future through direct measurement of SCFAs in fecal and blood samples.

We also observed several gut microbiota traits that may affect the health of centenarians. The enrichment of reported probiotics such as *Bifidobacterium* was detected in the centenarians. *B. adolescentis* and B. longum are the most abundant species belonging to *Bifidobacterium.* Noticeably, only *B. adolescentis* was highly enriched in the centenarians and young compared with the elderly in our cohort. *B. adolescentis* has been shown to directly influence Th17 cell generation (55). These interleukin 17 (IL-17)-producing T cells have a yin and yang effect on gut inflammation: on one hand, in their absence or in the absence of IL-17 signaling, gut dysbiosis is increased, but on the other hand, Th17 cells are associated with inflammatory bowel disease (IBD) and can exacerbate arthritis in mice (55, 56). The role *B. adolescentis* plays in the Th17-inflammation axis and mucosal immunity in centenarians is unknown. The significant enrichment of *B. adolescentis* in centenarians suggests a possible association between gut microbiota and inflammatory status in the gut of centenarians. Interestingly, a previous study has shown the high levels of proinflammatory cytokines IL-6 and IL-8 in centenarians (36).

*M. smithii* was reported to correlate with the consumption of milk products and perform specialized functions beneficial to the host (57, 58). Previous studies have shown that *M. smithii* can decrease the level of trimethylamine (TMA) which has been shown to correlate with clot-related events such as heart attacks and strokes (41, 59–61). Interestingly, coenzyme M- and F420-related gene families were significantly enriched in the centenarian group in our study; these enzymes are critical for decreasing TMA via methanogenesis (62). We also observed the enrichment of genes encoding components of the menaquinol biosynthesis and flavin biosynthesis pathways in centenarians. Menaquinol is important for bone and heart health (63–65), whereas riboflavin, an essential nutrient that cannot be synthesized by mammals, participates in a diversity of redox reactions central to human metabolism (66).

We found that the gut microbiota of centenarians have lower abundance of genes encoding components involved in the degradation of complex carbohydrates, which correlates with the significantly lower abundance of *F. prausnitzii*, *R. sp_5_1_39BFAA*, and *E. rectale* in the gut compared with the young and elderly. These three species are capable of utilizing complex carbohydrates in the gut (67). Our pathway analysis specifically showed a lower abundance of genes encoding components of pathways involved in degradation of dietary fiber such as starch, pectin, and cellulose. The fermentation of fibers in the gut starts with the breakdown of polysaccharides into simple carbohydrates that are used to produce pyruvate by glycolysis (68). Dietary fiber has been found to be critical for gut function (42, 67, 69). The long-term effect of low dietary fiber intake results in decreased microbiota diversity, decreased SCFA production, and gut barrier disruption (70–72). The poor capacity for fiber degradation in the centenarian gut microbiota suggests that the dietary fiber-deprived gut microbiota in an extreme-aging population may contribute to the risk of inflammation and gut barrier disruption (36, 73). Moreover, we found that the centenarian gut microbiota also has lower gene pathway abundance involved in the degradation of another carbohydrate, galactose. The decrease in galactose metabolism may contribute to the high incidence of cataracts frequently reported in centenarians (74–77). The impact of poor carbohydrate degradation capability on the health of centenarians is not clear at this time; however, future dietary intervention studies targeting nondigestible fibers may help better define health outcomes and diet in Sardinian centenarians.

**FIG 5:**
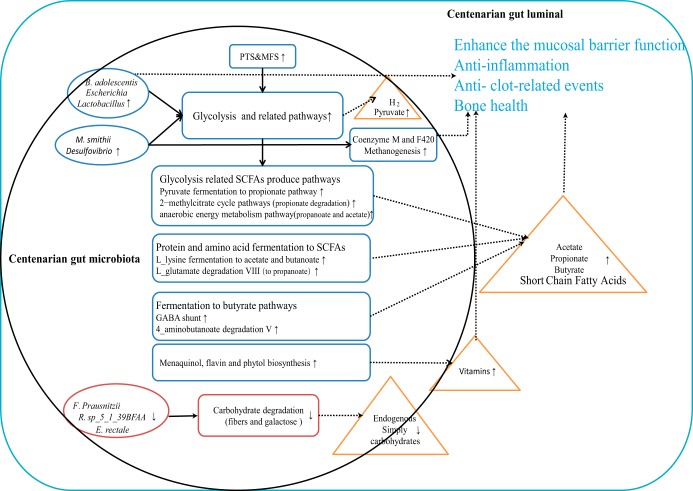
The working model of gut microbiota in Sardinia centenarian. The schematic diagram showing the compositional and functional features in the gut microbiota in Sardinian centenarians that had a predicted longevity association. Observed high abundance (red) or low abundance (blue) of compositional (ellipse) and functional (round rectangle) features are shown. Potential metabolites (in orange triangles) released by the gut microbes and proposed possible contributions of the gut microbes to metabolites (dotted curve) and metabolic functions (arrow) are shown.

Overall, our findings based on metagenomic data and previous studies support a working model (Fig. 5) where the centenarian gut microbiota is enhanced in genes encoding components involved in glycolysis and SCFA production, although the microbiota is deficient in carbohydrate degradation genes. The high abundance of *B. adolescentis*, *M. smithii*, *Escherichia*, and *Lactobacillus* along with the lower abundance of *F. prausnitzii*, *R. sp_5_1_39BFAA*, and *E*. *rectale* support this working model in taxonomic composition. In agreement with previous studies which underscored SCFAs as important gut microbiota metabolites in centenarians (20, 22, 23, 36), we hypothesize that increased SCFA production via glycolysis and related pathways as well as amino acid fermentation in the centenarian gut microbiota could be a pivotal beneficial factor for healthy aging and contribute to longevity.

How and when centenarians acquire their specific gut microbiota are still unknown. There are several factors that may contribute to the observed variations between centenarians and the young and elderly, such as host physiological decline, dietary changes, decline of immune function, increased inflammation, and genetics. Exercise, medication, lifestyle, and health status are all likely important factors as well. Diet is a pivotal factor that regulates the gut microbiota (5, 54, 78). In centenarians, diet could differ substantially from the diet of the young and elderly due to physiological changes. Reduced gastrointestinal tract function and a reduced ability to masticate certain foods may alter food preference and eating habits. A reduced ability to taste and smell may also alter food preferences (79, 80). Although our study lacks detailed dietary information, the Mini Nutritional Assessment (MNA) of centenarians showed a risk for malnutrition similar to what has been found in previous studies (81, 82). It is possible that a low capacity for carbohydrate degradation in centenarians reflects an adaptive consequence of gut microbiota under the influence of a long-term low-fiber diet. Inflammation related to aging (83), which can cause gut luminal oxygen levels to rise (84), may promote aerobic respiration in the gut. We observed aerobic respiration pathway enrichment in centenarians. Health status also acts as a covariate of the gut microbiota in centenarians. Previous research suggests that health status, including frailty and inflammatory status of the elderly, closely correlates with the composition of gut microbiota and diet (54, 85, 86), as the gut microbiota structures were similar in individuals with similar health status.

Our study analyzed the gut microbiota composition at the species level and metabolic function at the community level. Resolution at the strain level must be conducted to assess the contribution of the gut microbiota to metabolic function (87). Furthermore, surveying the gut microbiota by metagenomic sequencing of fecal samples had a limitation: we were unable to separate the viable microbes from the nonliving microbes. For example, the anaerobes that have different abundances in the colony and fecal material may be due to oxygen exposure during defecation and sample processing. Metatranscriptomics and metaproteomics can detect gut microbiota at the gene and protein expression levels (88). Such multi-omics approaches will be integrated into further studies. Last, if we want to demonstrate the causative role the strains play in longevity, we should perform follow-up mechanistic studies with different environmental and dietary conditions in defined animal models.

In summary, the taxonomic and functional profiles we observed in the gut microbiota in Sardinian centenarians revealed the complex and adaptable nature of the gut ecosystem. The gut microbiota in Sardinian centenarians display the potential health-promoting signatures that are involved in the high capability in glycolysis and SCFA production, which could boost longevity, and also show the aging-related “inflammation” trails that may relate to low capability in complex carbohydrate degradation which could be maladaptive to the extreme aging. Our study here represents a useful and important expansion of previous research investigating the gut microbiota in centenarians, highlighting the possible features of gut microbiota that could identify important health-related function in Sardinian centenarians, providing new prospective targets for gut microbiota and host physiology research in the future.

## MATERIALS AND METHODS

### Subject recruitment and clinical information collection.

We recruited 65 subjects in Sardinia, Italy, as part of the AKEntAnnos (AKEA) project that is studying the extreme longevity in Sardinia (24). Ethical approval was provided by the Institutional Local Ethics Committee, Azienda Sanitaria Locale n.1 of Sassari, Italy. The donors were volunteers recruited from the longevity AKEA project, and participants gave written consent. Subjects were divided into three age groups: young, elderly, and centenarian. Exclusion criteria for the young group and the elderly group included the following: (i)history of chronic medical conditions (diabetes, hypertension) and (ii) use of antimicrobial medication (antibiotic or antifungal treatments) 1 year before sampling. Clinical history, medical history, and anthropometric measurements were collected based on the self-report (for elderly) and health care numbers (for centenarians). The clinical and nutritional data were collected as described in the AKEA study (24). MNA to assess malnutrition, MMSE to evaluate cognitive status, and FIM to assess disability and healthy parameter records were also recorded.

### Sample collection and DNA extraction.

Fecal samples were collected by the participants at home. Participants were provided with a stool specimen collection tube. After the study participant passed stools, the participant used a spoon to collect about 1 g stool sample by scraping off the outer layer of solid feces and collecting the central part into the tube. Samples were immediately frozen at home at −20°C and collected by laboratory personnel within 6 weeks. Long-term storage of samples was in −80°C freezers located at the University of Sassari. Stool metagenomic DNA was extracted according to the manual instructions for the QIAamp DNA Stool Mini kit (Qiagen) with some modifications. Briefly, 200 mg of stool was suspended in 1.4 ml of ASL buffer and 0.4 g of 5-mm zirconia beads (Sigma) was added. Then each sample was subjected to a bead beating step using Biosan for a maximum of 3,000 rpm for 30 min. Samples were heated at 95°C for 5 min and then centrifuged for 5 min at 13,000 rpm to pellet stool particles. Next, 1.2-ml supernatants were collected, and the InhibitEX tablet was added, followed by incubation at room temperature (RT) for 1 min and centrifugation at 13,000 rpm for 3 min; then 15 μl proteinase K and 200 μl AL buffer was added to 200 μl supernatant and incubated at 70°C for 10 min. Two hundred microliters of absolute ethanol was then added to the mixture, vortexed, and loaded on QIAamp Mini spin columns. The columns were washed with AW1 and AW2 buffer per the QIAamp DNA Stool Mini kit instructions. The DNA was eluted with 200 μl TE buffer. Finally, the DNA concentration was determined by using NanoDrop ND-1000 (NanoDrop Technologies).

### Shotgun metagenomic sequencing.

Illumina libraries were prepared with 100 ng of input DNA, using KAPA Hyper Prep kit (Kapa Biosystems) following the manufacturer’s instructions. Libraries were quality checked by KAPA Library Quantification kit (Kapa Biosystems) and 2100 Bioanalyzer (Agilent). The qualified Illumina libraries were then transported on dry ice to BGI-Shenzhen for paired-end metagenomic sequencing which was performed on an Illumina HiSeq X10 PE150 platform (with an average insert size of 350 bp). The sequence reads were first filtered by the in-house pipeline at BGI-Shenzhen. A total of 6.2 × 10^9^ clean reads (Q20 percentage 95%) were generated for 59 samples.

### Bioinformatics and statistical analysis.

**(i) Bioinformatics for shotgun metagenomic sequencing.** Clean reads were mapped against the human genome (hg19) with BWA (version 0.7.12) to remove human contamination (89). The filtered, clean reads were used as input for further analysis. The profiles of microbiota composition were predicted using MetaPhlan2.0, and gene family profiles and pathway profiles were predicted using HUMANN2 with default parameters (30, 90). The gene family profile was normalized by reads per kilobase, annotated to the UniProt Reference Cluster (UniRef90). Further pathway mapping and regrouping were performed using the MetaCyc metabolic pathway database. The gene family profile was regrouped to the orthologous groups using the KEGG database. Gene pathways were calculated from the constituent gene family abundance for each individual. We also used the integrated catalog of reference genes in the human gut microbiome (IGC) as a reference for mapping our clean reads; the IGC gene catalog is a published, high-quality reference catalog generated from thousands of subjects around the world (31). BWA (version 0.7.12) was used for the mapping of the clean reads to the IGC gene catalog. Samtools (version 0.1.19) was used to determine the matching results (31). On average, 74% of sequencing reads successfully mapped to the IGC database, with 2.5 × 10^7^ properly paired reads for each subject. The annotation results were compared with HUMANN and IGC methods to validate data analysis.

**(ii) Statistical analysis.** All statistical analyses were performed using R software (version 3.4.2). Multivariate analyses of community diversity, including PCoAs and NMDS were performed using ade4 and vegan (version 2.5-1) and visualized using ggplot2 and ggpubr. The Shannon diversity index and species richness were calculated using the same package. The Bray-Curtis distance matrix was used as a similarity index. Hierarchical clustering method in heatmap is complete, and the distance matrix is created by Pearson correlation. ANOVA followed by the Tukey-Kramer multiple-comparison test or Kruskal-Wallis test followed by Dunn’s post-hoc multiple-comparison test were used to determine whether significant differences existed between multiple groups (91). Welch’s two-sided *t* test was used for the analysis of variances of two groups. Similarities among groups were detected by MRPP and ANOSIM methods using 999 permutations to test the significance. The MRPP statistic delta is simply the overall weighted mean of within-group means of the pairwise dissimilarities among each age group. The effect size and significance of covariate (referred to genus and healthy parameters, respectively, in Fig. 1 and 4) were determined by the envfit function in vegan. Ordination was performed using PCoAs based on Bray-Curtis dissimilarity. The significance value was determined based on 999 permutations.

### Data availability.

All data are available in ENA under study accession number PRJEB25514.
