# Supplementary material for: A Cross-Sectional Study of Compositional and Functional Profiles of Gut Microbiota in Sardinian Centenarians
Source: mSystems. 2019 Jul 9;4(4):e00325-19. doi: 10.1128/mSystems.00325-19 (PMC6616150; doi:10.1128/mSystems.00325-19)
Supplement: TABLE S1 [file mSystems.00325-19-st001.docx]

| Table S1 Full demographical and clinical information for each subject. | | | | | | | | | | |
| --- | --- | --- | --- | --- | --- | --- | --- | --- | --- | --- |
| #SampleID | Gender | Group | Age | MMSE | MNA | Drug | BMI | FIM_M | FMI_C | FIM |
| C_6G16 | M | C | 102 | 23 | 22 | 1 | 27.8 | 91 | 32 | 123 |
| C_7G27 | F | C | 101 | 26 | 23 | 0 | 19.1 | 91 | 31 | 122 |
| C_7G1 | F | C | 102 | 20 | 20 | 5 | 24.2 | 77 | 31 | 108 |
| C_7G5 | F | C | 102 | 19 | 8 | 3 | 22.2 | 75 | 31 | 106 |
| C_7G51 | F | C | 100 | 26 | 16.5 | 5 | 28.1 | 57 | 35 | 92 |
| C_7G4 | M | C | 99 | 6 | 13.5 | 7 | 20.5 | 73 | 18 | 91 |
| C_6G6 | F | C | 104 | 12 | 18 | 3 | 23.8 | 68 | 20 | 88 |
| C_7G8 | M | C | 103 | 20 | 26 | 2 | 24.4 | 57 | 30 | 87 |
| C_7G6 | M | C | 100 | 26 | 25 | 5 | 21.7 | 44 | 35 | 79 |
| C_7G28 | M | C | 101 | 23 | 18.5 | 4 | 24.2 | 57 | 22 | 79 |
| C_7G32 | F | C | 100 | 5 | 23.5 | 3 | 26.7 | 55 | 20 | 75 |
| C_7G3 | M | C | 107 | 13 | 21 | 7 | 24.2 | 58 | 15 | 73 |
| C_7G31 | F | C | 100 | 14 | 20.5 | 3 | 26.1 | 42 | 23 | 65 |
| C_6G13 | F | C | 102 | 6 | 17 | 5 | 24.6 | 48 | 15 | 63 |
| C_6G15 | F | C | 102 | 6 | 17 | 3 | 23.5 | 48 | 13 | 61 |
| C_6G14 | F | C | 102 | 20 | 24 | 6 | 24.3 | 32 | 18 | 50 |
| C_6G8 | F | C | 106 | 9 | 15 | 5 | 23.3 | 32 | 13 | 45 |
| C_7G2 | F | C | 101 | 19 | 14 | 5 | 17.9 | 21 | 13 | 34 |
| C_6G10 | F | C | 101 | 8 | 17 | 3 | 20.2 | 22 | 9 | 31 |
| E_7G29 | M | E | 73 | 30 | 28 | 4 | 25 | 91 | 35 | 126 |
| E_7G45 | M | E | 68 | 30 | 27 | 1 | 36.9 | 91 | 32 | 123 |
| E_7G43 | M | E | 81 | 27 | 27 | 5 | 30.4 | 91 | 32 | 123 |
| E_7G30 | M | E | 70 | 25 | 27 | 1 | 24.8 | 91 | 35 | 126 |
| E_7G42 | F | E | 74 | 27 | 26.5 | 1 | 19.9 | 91 | 32 | 123 |
| E_7G40 | F | E | 68 | 30 | 26 | 2 | 32.8 | 91 | 35 | 126 |
| E_7G49 | M | E | 69 | 28 | 26 | 0 | 26.4 | 91 | 35 | 126 |
| E_7G47 | M | E | 71 | 30 | 25.5 | 1 | 35.8 | 91 | 35 | 126 |
| E_7G21 | F | E | 85 | 24 | 24.5 | 0 | 29.1 | 91 | 34 | 125 |
| E_7G22 | F | E | 82 | 22 | 24.5 | 6 | 22.7 | 91 | 30 | 121 |
| E_7G24 | M | E | 88 | 23 | 24 | 9 | 26.4 | 91 | 32 | 123 |
| E_7G41 | F | E | 76 | 30 | 24 | 3 | 22.6 | 91 | 35 | 126 |
| E_7G38 | M | E | 75 | 28 | 24 | 0 | 21.5 | 91 | 33 | 124 |
| E_7G44 | M | E | 69 | 30 | 23.5 | 1 | 31.5 | 91 | 35 | 126 |
| E_7G26 | F | E | 78 | 24 | 23.5 | 3 | 25.6 | 91 | 30 | 121 |
| E_6G7 | F | E | 85 | 23 | 22.5 | 3 | 19.9 | 91 | 28 | 119 |
| E_7G25 | F | E | 78 | 20 | 22 | 11 | 20.4 | 91 | 31 | 122 |
| E_7G46 | F | E | 69 | 30 | 22 | 0 | 19.5 | 91 | 32 | 123 |
| E_7G20 | F | E | 83 | 23 | 21 | 1 | 24.6 | 91 | 32 | 123 |
| E_7G48 | F | E | 84 | 30 | 20.5 | 5 | 26.3 | 91 | 35 | 126 |
| E_7G7 | F | E | 85 | 24 | 18 | 5 | 21.2 | 89 | 31 | 120 |
| Y_6G1 | F | Y | 23 | * | * | 0 | 19.0 | * | * | * |
| Y_6G11 | F | Y | 22 | * | * | 0 | 19.1 | * | * | * |
| Y_6G12 | M | Y | 22 | * | * | 0 | 23.0 | * | * | * |
| Y_6G2 | F | Y | 22 | * | * | 0 | 20.0 | * | * | * |
| Y_6G3 | M | Y | 22 | * | * | 0 | 21.9 | * | * | * |
| Y_6G4 | M | Y | 21 | * | * | 0 | 22.9 | * | * | * |
| Y_6G5 | M | Y | 30 | * | * | 0 | 24.6 | * | * | * |
| Y_7G12 | F | Y | 31 | * | * | 0 | 22.3 | * | * | * |
| Y_7G14 | M | Y | 27 | * | 25.5 | 0 | 24.5 | * | * | * |
| Y_7G15 | F | Y | 27 | * | 24 | 0 | 26.3 | * | * | * |
| Y_7G16 | F | Y | 33 | * | 25.5 | 0 | 25.2 | * | * | * |
| Y_7G17 | F | Y | 25 | * | 26.5 | 0 | 17.4 | * | * | * |
| Y_7G18 | F | Y | 30 | * | 22 | 0 | 16.2 | * | * | * |
| Y_7G19 | M | Y | 21 | * | 28 | 0 | 20.7 | * | * | * |
| Y_7G33 | F | Y | 24 | * | 20.5 | 0 | 17.6 | * | * | * |
| Y_7G34 | F | Y | 23 | * | 27 | 0 | 40.1 | * | * | * |
| Y_7G39 | M | Y | 30 | * | 22.5 | 0 | 26.9896 | * | * | * |
| E_7G10 | F | E | 80 | * | * | * | * | * | * | * |
| E_7G50 | M | E | 85 | * | * | * | * | * | * | * |
| * unmeasured | |  |  |  |  |  |  |  |  |  |
| Group: C:centenarian group; E:healthy elderly group; Y: healthy young group | | | | | | | | |  |  |
| BMI: Body Mass index | | |  |  |  |  |  |  |  |  |
| MMSE: Mini-Mental State Examination | | | | |  |  |  |  |  |  |
| MNA: Mini Nutritional Assessment; | | | |  |  |  |  |  |  |  |
| FIM: Functional Independence Measure | | | | |  |  |  |  |  |  |
| FIM-C: Functional Independence Measure-Congnitive | | | | | |  |  |  |  |  |
| FIM-M: Functional Independence Measure-Motor | | | | | |  |  |  |  |  |
| Drug: The intake of medicine in each subject | | | | |  |  |  |  |  |  |
