# Supplementary material for: A Cross-Sectional Study of Compositional and Functional Profiles of Gut Microbiota in Sardinian Centenarians
Source: mSystems. 2019 Jul 9;4(4):e00325-19. doi: 10.1128/mSystems.00325-19 (PMC6616150; doi:10.1128/mSystems.00325-19)
Supplement: TABLE S2 [file mSystems.00325-19-st002.docx]

| Table S2 Statistically variation between the demographical and clinical information in different age groups | | | | |
| --- | --- | --- | --- | --- |
| One-way ANOVA followed by Tukey's post-hoc multiple comparisons | Age | MNA | Drug | BMI |
|  |  |  |  |  |
| C vs. E | **** | **** | NO | NO |
| C vs. Y | **** | *** | **** | NO |
| E vs. Y | **** | NO | *** | NO |
|  |  |  |  |  |
| T-test | MMSE | FIM-M | FMI-C | FIM |
| C vs. E | *** | **** | **** | **** |
| ∗ P < 0.05; ∗∗ P < 0.01; ∗∗∗ P < 0.001;∗∗∗∗ P < 0.0001 | |  |  |  |
| Group: C:centenarian group; E:healthy elderly group; Y: healthy young group | | | |  |
| BMI: Body Mass index |  |  |  |  |
| MMSE: Mini-Mental State Examination |  |  |  |  |
| MNA: Mini Nutritional Assessment; |  |  |  |  |
| FIM: Functional Independence Measure |  |  |  |  |
| FIM-C: Functional Independence Measure-Congnitive | |  |  |  |
| FIM-M: Functional Independence Measure-Motor | |  |  |  |
| Drug: The intake of medicine in each subject |  |  |  |  |
